# Supplementary material for: Splice-Junction-Based Mapping of Alternative Isoforms in the Human Proteome
Source: Cell Rep. Author manuscript; Available in PMC 2020 Jan 15. (PMC6961840; doi:10.1016/j.celrep.2019.11.026)
Supplement: 2 [file NIHMS1546469-supplement-2.zip › DF1/PXD010154/Liver/Liver_66_DVL1_GPSASLWPSAGTQRPEATSPSHGVSGPR.pdf]

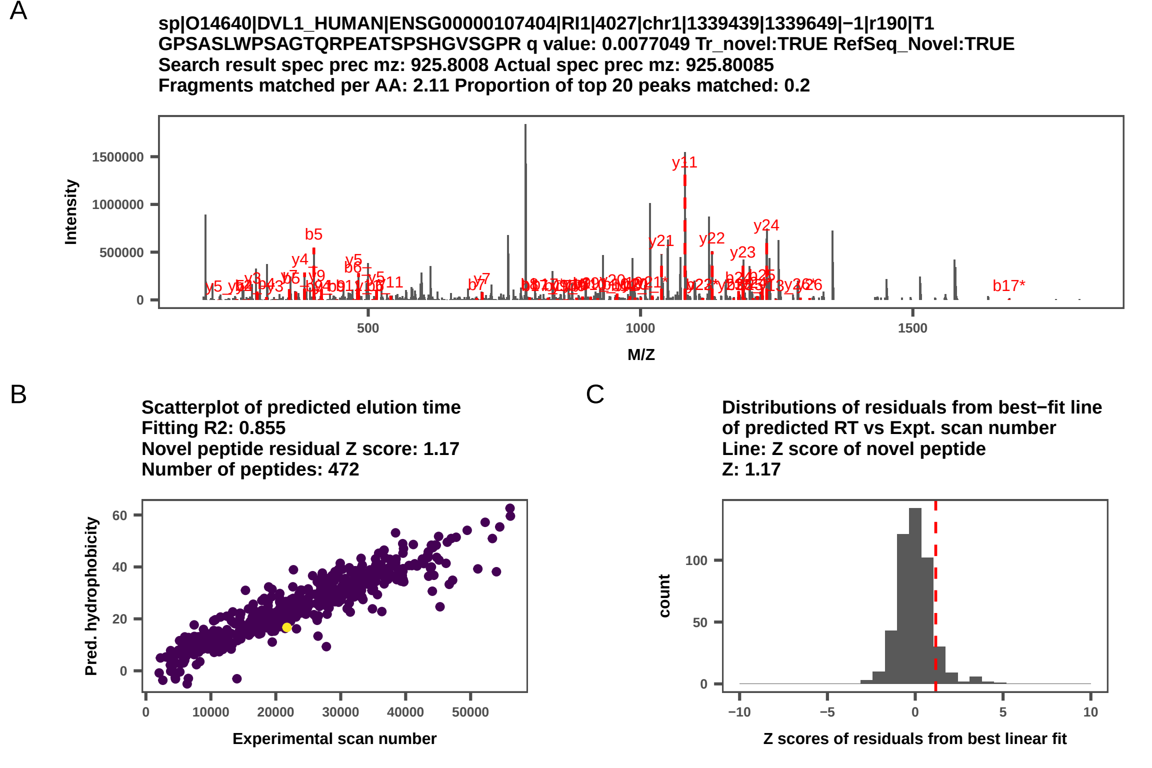
A
sp|O14640|DVL1_HUMAN|ENSG00000107404|RI1|4027|chr1|1339439|1339649|−1|r190|T1
GPSASLWPSAGTQRPEATSPSHGVSGPR q value: 0.0077049 Tr_novel:TRUE RefSeq_Novel:TRUE
Search result spec prec mz: 925.8008 Actual spec prec mz: 925.80085
Fragments matched per AA: 2.11 Proportion of top 20 peaks matched: 0.2
Intensity
1500000
1000000
500000
0
y11
b5
y24
y21
y22
y23
y4
b7
y7
b17*
500
1000
1500
M/Z
B
Scatterplot of predicted elution time
Fitting R2: 0.855
Novel peptide residual Z score: 1.17
Number of peptides: 472
Pred. hydrophobicity
60
40
20
0
0
10000
20000
30000
40000
50000
Experimental scan number
C
Distributions of residuals from best−fit line
of predicted RT vs Expt. scan number
Line: Z score of novel peptide
Z: 1.17
count
100
50
0
−10
−5
0
5
10
Z scores of residuals from best linear fit
